# Reverse reaming distraction for acetabular reconstruction of chronic pelvic discontinuity

**DOI:** 10.1186/s13018-020-01701-x

**Published:** 2020-05-24

**Authors:** Jing-yang Sun, Ming Ni, Hai-yang Ma, Yin-qiao Du, Jun-min Shen, Ji-ying Chen, Yong-gang Zhou, Guo-qiang Zhang

**Affiliations:** 1 Medical School of Chinese People’s Liberation Army General Hospital, Beijing, 100853 China; 2 Department of Orthopedics, the First Medical Center, Chinese People’s Liberation Army General Hospital, Beijing, 100853 China

**Keywords:** Pelvic discontinuity, Total hip arthroplasty, Acetabular distraction, Reverse reaming, Revision

## Abstract

**Background:**

The acetabular distraction technique demonstrates encouraging radiographic and clinical outcomes in treating chronic pelvic discontinuity. The aim of this study is to describe a modified distraction technique and to show our results.

**Methods:**

This study identified 12 cases of chronic pelvic discontinuity undergoing primary or revision total hip arthroplasty (THA) with the technique of reverse reaming distraction between July 2015 and November 2018. All 12 patients had a minimum follow-up of 12 months. Radiographs were reviewed to inspect for component loosening. Clinical assessment included the Harris hip score (HHS) and an ambulatory scoring system.

**Results:**

At the time of final follow-up, no patient was revised. One patient had up to 1 cm migration of the cup in a horizontal or vertical direction and more than 20° change in the abduction angle but was asymptomatic. In the remaining 11 patients, no migration of the component was detected. Both the HHS and ambulatory score showed improvement in all patients. There were no perioperative complications. No postoperative dislocation occurred.

**Conclusions:**

Reverse reaming distraction is a feasible technique in treatment of chronic pelvic discontinuity, with encouraging results at early term. However, ongoing follow-up is required to determine the long-term prognosis in patients receiving this technique.

## Background

Pelvic discontinuity is an uncommon problem in acetabular revisions or in complex primary THAs, which is defined by the absence of bridging bone between the superior and inferior hemipelvis [1]. Due to substantial bone loss associated with pelvic discontinuity, the acetabular reconstruction remains a challenging task.

Several options are available for treating pelvic discontinuity. Acute pelvic discontinuity is amenable to posterior column compression plating and implantation of conventional hemispherical acetabular cup [2]. Chronic pelvic discontinuity is often secondary to chronic bone loss due to osteolysis and loosening of an acetabular component [3], in which the residual bone has a poor capability of stabilizing the acetabular component and healing the discontinuity. The main management options for chronic pelvic discontinuity include structural allograft with a cage, cup-cage construct, custom triflange acetabular component (CTAC), or acetabular distraction technique [4].

The midterm failure of using a major column structural allograft combined with a reconstruction cage has been widely reported. It is attributed to the absence of permanent biological fixation. There are concerns about pull-out or breakage of the flanges, allograft resorption, and infection [5, 6]. Consequently, the use of structural allografts has been decreasing over the last decade, and alternative reconstructive measures with cup-cage construct have been preferred, which can provide sufficient primary mechanical stability and potential of subsequent biological bone in-growth. Martin et al. [7] retrospectively analyzed four different modalities for the treatment of pelvic discontinuity and found the 5-year revision-free survivorship of the implant was best with a cup-cage construct (100%). Moreover, a CTAC based on three-dimensional (3D) CT scan can also address acetabular defects with a chronic pelvic discontinuity. However, the considerably high complication rates as literatures demonstrated limit its extensive application [8].

Acetabular distraction technique, first described at length by Sporer et al. [9], demonstrates encouraging clinical and radiographic outcomes [10]. However, to our knowledge, there have been few reports with respect to its use in other institutions than theirs. From July 2015 to November 2018, we have treated 12 chronic pelvic discontinuities by a modified distraction technique. Different from their technique, we did not use a dedicated extra-acetabular distractor. The distraction of pelvis can also be accomplished by reverse reaming with incremental size, which produces a centrifugally impacting force. In this study, we described our experience and report the outcomes associated with this technique.

## Patients and methods

Institutional Review Board approval was obtained. This study identified 12 cases of chronic pelvic discontinuity undergoing primary or revision THA with the technique of reverse reaming distraction between July 2015 and November 2018. All 12 patients had a minimum follow-up of 12 months (average, 24 months; range, 12–52 months). Within this cohort, the initial diagnosis was osteoarthritis in 1, osteonecrosis in 7, post-traumatic arthritis in 1, pathological acetabular fracture after pelvic irradiation for vaginal cancer in 1, and developmental dysplasia of the hip in 2. Before index operation, three patients had a history of periprosthetic joint infection, and one patient had a history of glucocorticoid pulse therapy for encephalitis. We evaluated preoperative radiographs according to the Paprosky classification [11]. Pelvic discontinuity in all cases was identified preoperatively by 3D printing model. Demographic data were summarized (Table 1).

**Table 1:** Demographics

Case	Initial diagnoses	Gender	Age (year, index procedure)	Height (cm)	Weight (kg)	BMI (kg/cm^2^)	Number of previous surgeries	Paprosky classification	Failed acetabular fixation
1	DDH; sequelae of poliomyelitis	F	51	165	60	22.0	1	IIIB	Uncemented
2	Pathological acetabular fracture after pelvic irradiation	F	58	168	90	31.9	0	–	–
3	Osteonecrosis	F	63	175	75	24.5	2	IIIB	Uncemented
4	Osteonecrosis	F	53	155	80	33.3	2	IIIA	Cemented
5	Post-traumatic arthritis; acetabular fracture non-union	M	57	168	70	24.8	0	–	–
6	Osteonecrosis	F	75	160	70	27.3	2	IIIB	Uncemented
7	Osteoarthritis	F	68	158	50	20.0	1	IIIB	Cemented
8	DDH	F	37	163	53	21.1	1	IIC	Uncemented
9	Osteonecrosis	F	66	156	70	28.8	2	IIIB	Uncemented
10	Osteonecrosis	M	64	170	80	27.7	3	IIIB	Uncemented
11	Osteonecrosis	F	57	158	62	24.8	2	IIC	Uncemented
12	Osteonecrosis	M	55	176	80	25.8	2	IIIB	Uncemented

### Surgical techniques

**Fig. 1:**
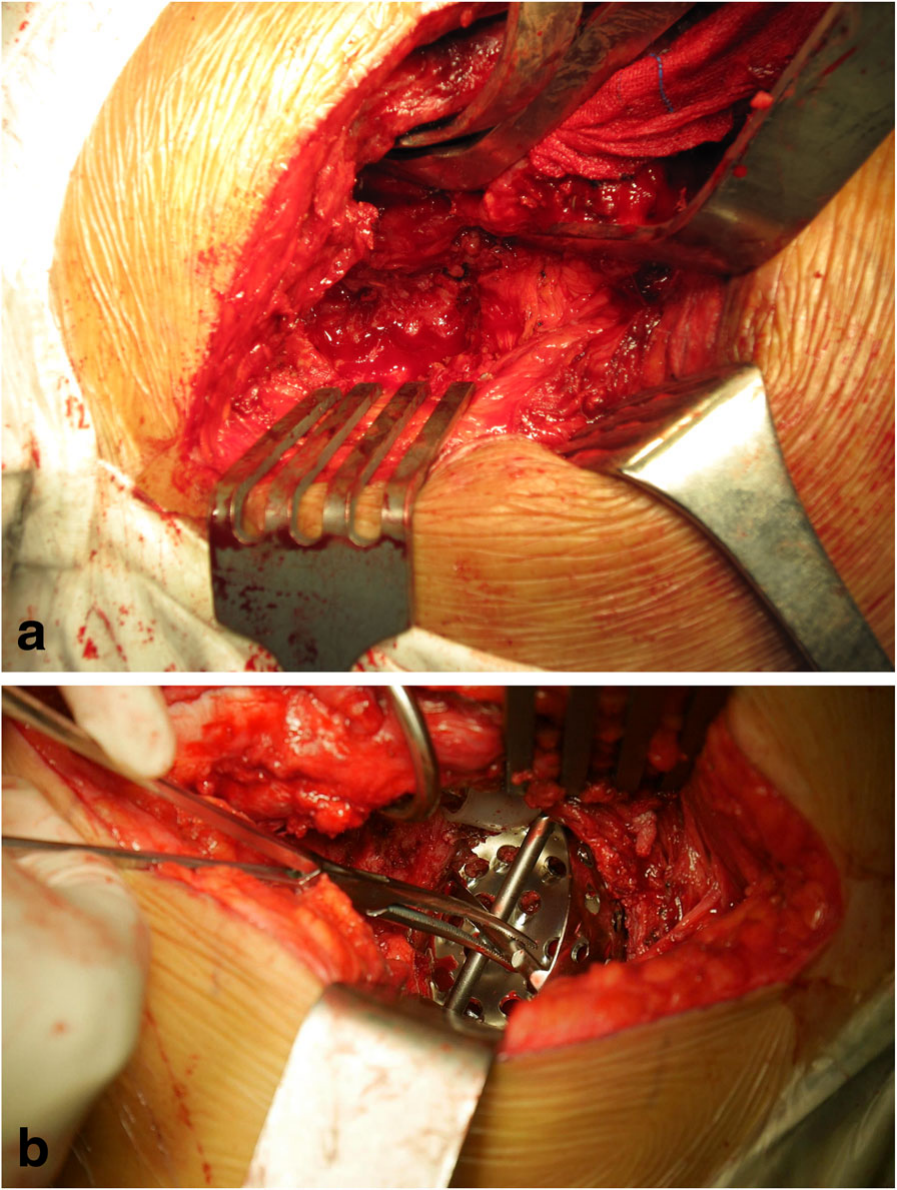
**a** Intraoperative confirmation of the pelvic discontinuity. **b** The acetabulum engaged by the reamer and moved as a unit

Surgeries were performed by two of the authors through a posterolateral approach. After exposure of the acetabulum, the discontinuity was confirmed by demonstrating movement between the superior and inferior halves of the pelvis. Granulation tissue and any debris were cleaned to allow an accurate evaluation of the bone stock. Gentle reaming of the acetabulum was performed at the anatomical level to minimize the risk of removing a significant amount of remaining host bone. When the fossa showed a round shape as a whole, reverse reaming with an incremental size was performed with the aim of distracting the fibrous tissue around the acetabulum. The appropriate-sized reamer was defined as enough to keep it and its unsupported handle in place, as well as allowing the surgeon to move the patient’s pelvis as a unit by moving the handle. With severe bone defects, the reamer may not fully engage some specific regions of the acetabulum. In this situation, modular trial augments were placed within the defect to determine the optimal augment configuration and size that would support the acetabular component. After contouring the defect with a barrel burr to optimize the surface contact area, augment implant was secured to the host bone with one or two screws. Again, reverse reaming was performed to distract the acetabulum, and the appropriate size was redetermined (Fig. 1).

The acetabular component chosen for implantation was the same size as the last reamer. With the large-diameter, porous metal acetabular component acting as an “internal plate,” the discontinuity could be bridged and treated in distraction. Through the acetabular cup, multiple cancellous screws could be placed into the remaining ilium and ischium. Cement was used to bond the augment and the acetabular component. The use of additional overlying cage was left to the surgeon’s discretion about the extent of osteoporosis, bone erosion caused by previous infection, and weak muscle strength of contralateral limb as a result of sequela of poliomyelitis.

Postoperatively, all patients were allowed touchdown weight-bearing with a walking aid device for the first 12 weeks and then gradual progression to bearing full weight as tolerated. Anticoagulation therapy was generally administered for 4 weeks. Two patients undergoing primary THA received parenteral antibiotics for 24 h. Seven patients revised for aseptic loosening received parenteral antibiotics for 5 days followed by another 7 days of oral antibiotics. Three patients undergoing staged treatment of prosthetic joint infection were administered parenteral antibiotics for 4 weeks followed by oral antibiotics for 8 weeks.

### Postoperative assessment

All patients were evaluated clinically and radiographically before surgery, at 3, 6, and 12 months postoperatively and yearly thereafter. Clinical assessment included the Harris hip score [12], which was available for all patients preoperatively and at the last follow-up visit. We also used a modified ambulatory scoring system to evaluate the patients’ ability to walk, where a score of 1 indicates independent walking and a score of 6 indicates the use of a wheelchair [13].

The most recent follow-up radiographs were compared with the initial postoperative and serial radiographs. Independent review of the anteroposterior and lateral radiographs was performed by two reviewers to inspect for the presence of radiolucent lines, migration of the component, component loosening, and radiographic evidence of bony callus formation across the discontinuity. Acetabular component loosening was defined as greater than 10° change in the abduction angle or a change in the horizontal or vertical position of the hip center of more than 6 mm after correction for magnification [14]. Loosening of the cage and augments was determined according to Gill’s criteria [15] and the measure described by Abolghasemian et al. [13].

Definite or probable loosening of any acetabular component was a radiological failure. Due to the observation that some component migrates early after the surgery but eventually stabilize, we considered progressive migration of the component occurring after 1 year to be a radiological failure. Failure was defined as revision of the acetabular component due to any cause, HHS improvement of less than 20 points after the surgery, or radiological failure.

Harris score was expressed as mean ± SD (standard deviation). The pre- and postoperative HHS scores and ambulatory scores were compared using a paired Student’s *t* test and Wilcoxon’s matched-pairs signed ranks test. A *P* value < 0.05 denoted statistical significance. All data analysis was performed on the SPSS 21.0 software.

## Results

**Fig. 2:**
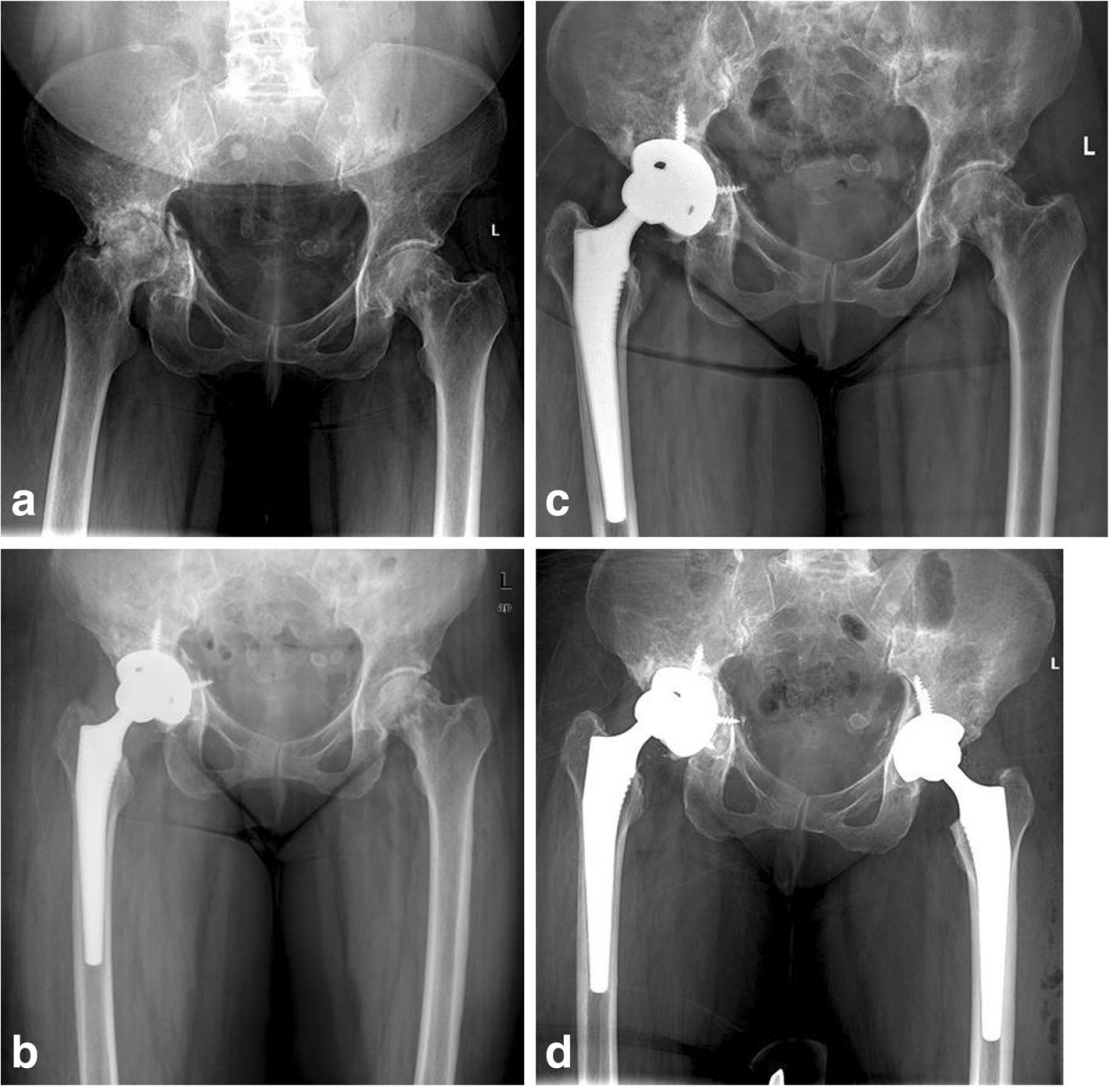
Serial radiographs of a patient with pathological acetabular fracture after pelvic irradiation for vaginal cancer. **a** Preoperative X-ray. **b** Immediate X-ray after surgery. **c** Migration of the cup observed at 1.5 years after surgery. **d** No intervention of the loosened cup due to no symptom and total hip arthroplasty of contralateral hip

**Fig. 3:**
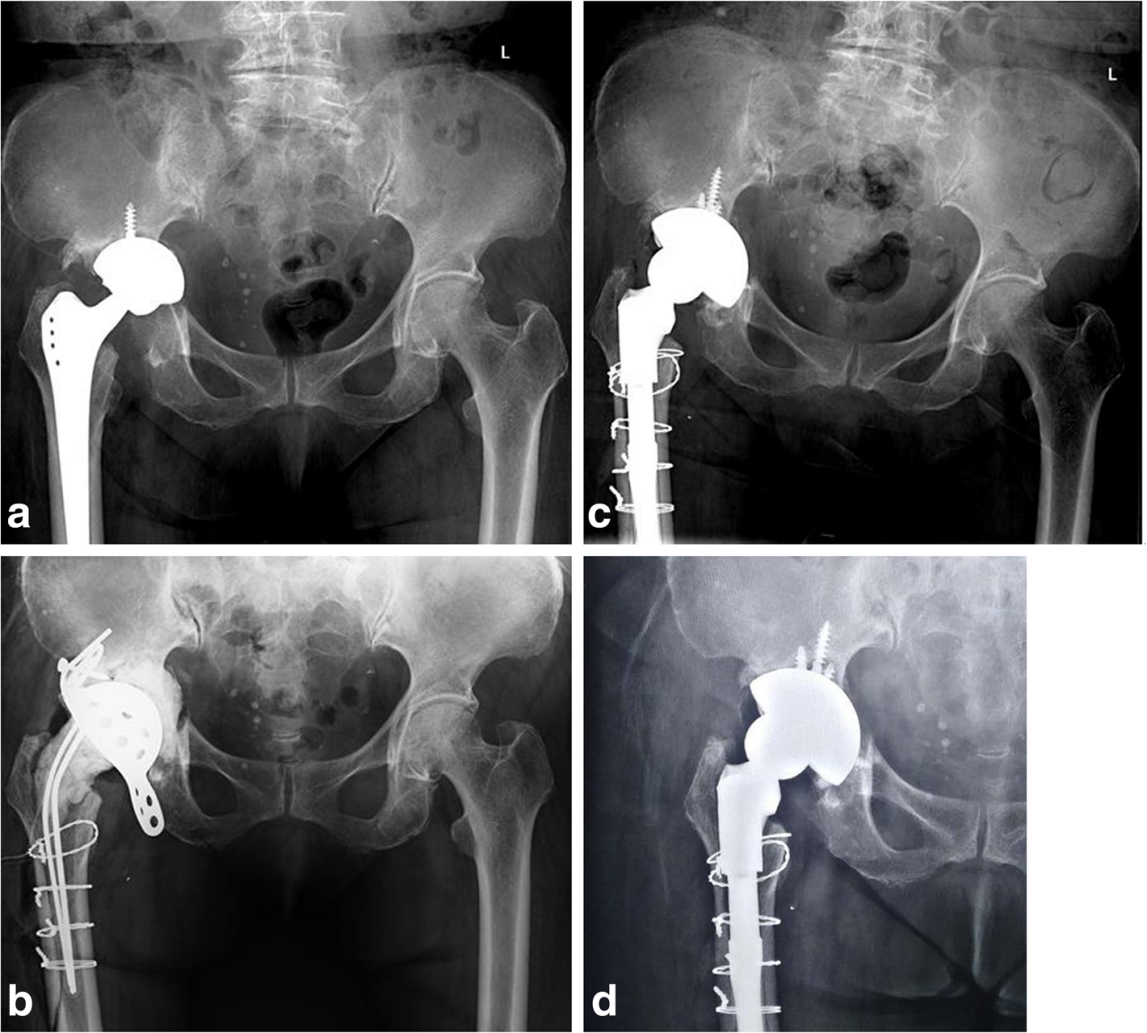
Serial radiographs of a patient of chronic pelvic discontinuity after periprosthetic joint infection. **a** Preoperative X-ray. **b** Implantation of a temporary spacer and anti-protrusion ring. **c** Immediate X-ray after second-stage reimplantation. **d** X-ray at 1 year after surgery

In 4/10 patients, the femoral component was revised because of aseptic or septic loosening. Liner with elevated rim was used in 8/12 patients. At the time of final follow-up, no patient was revised. One patient had up to 1 cm migration of the cup in a horizontal or vertical direction and more than 20° change in the abduction angle but was asymptomatic. The patient elected to continue without further intervention (Fig. 2). In the remaining 11 patients, no radiolucent lines, migration of the component, or component loosening was detected (Fig. 3).

Clinically, eight patients denied pain in the operative hip. Four patients reported minimal pain after walking more than 1 km. Two patients recovered to a normal gait without assistive devices, four patients showed slight limp but without need for assistive devices, four patients who reported pain after walking more than 1 km used one crutch for longer distances, one patient used two crutches at all times due to sequela of poliomyelitis and knee valgus deformity of the contralateral limb, and the patient with a migrated cup used one crutch to avoid excessive weight-bearing. None of the patients used a wheelchair. The improvement of the HHS was 54.5 ± 9.3 (19.9 ± 7.2 preoperatively to 74.5 ± 9.7 postoperatively) (*P* < 0.001). The mean ambulatory score preoperatively was 5.75 (5 to 6), which improved to 2.17 (1 to 5) (*P* < 0.001). There were no perioperative complications. No postoperative dislocation occurred (Table 2).

**Table 2:** Clinical data and outcomes

Case	Type of revision construct	Follow-up (months)	Harris hip score (points)		Ambulatory score (points)	
			Pre-op	Post-op	Pre-op	Post-op
1	Tantalum cup (64 mm) Cup-cage Moserllized allograft	38	15	60	6	5
2	Tantalum cup (56 mm)	41	16	64	6	3
3	Tantalum cup (66 mm) Cup-cage 3D-printed titanium augment	12	13.5	72	6	3
4	Tantalum cup (60 mm) Cup-cage	13	15	72	6	3
5	Tantalum cup (64 mm)	52	30	90	5	1
6	Tantalum cup (60 mm)	14	28	81	6	1
7	Pinnacle Gription cup (72 mm)	17	21.5	65	6	3
8	Tantalum cup (56 mm) Cup-cage	33	32	82	5	1
9	Pinnacle Gription cup (72 mm)	28	22	67	6	3
10	Pinnacle Gription cup (72 mm) Gription TF augment	19	14	89	6	1
11	Pinnacle Gription cup (66 mm)	17	22	77	5	1
12	Tantalum cup (70 mm) Cup-cage	13	10	75	6	1

## Discussion

There have been various reports of structural or cancellous allograft with acetabular cages in the treatment of chronic pelvic discontinuity [5, 6, 16]. On the whole, literatures indicate mechanical constructs like cages appear to provide unreliable outcomes, given the potential for fatigue and late loosening. Therefore, it is critical to achieve initial mechanical stability for bone ingrowth to occur into the prosthesis both superiorly and inferiorly to bridge the discontinuity in a biologic fashion. Both cup-cage construct and CTAC showed promising survivorships [6, 8]. However, the extensive soft tissue dissection necessary for implantation of these constructs may predispose the hip to instability and increase the risk of deep infection and nerve injury. The jumbo cup combined with acetabular distraction technique, by contrast, may be a more feasible option.

Only one of our 12 patients treated with the acetabular distraction technique required revision due to acetabular component loosening. Similarly, early migration of the acetabular component was encountered in the study by Sheth et al. [10]. They achieved stability in a new position and remained pain free. The loosed cup in our study migrated to a more steep orientation. Asymptomatic though she is, she has to face the high risk of accelerated wear and dislocation. The failure of ingrowth of the acetabular cup might be attributed to the killing effect of pelvic radiation. According to the short-term results by Berry et al. [17], structural allograft with cage reconstruction was more suitable for irradiated bone than cementless cup. Thus, it can be inferred that adequate contact with live host bone is the prerequisite of application of jumbo cup with acetabular distraction. No migration of the component was detected in the remaining patients. In a two-center radiological analysis by Sheth et al. [10], 69% (22/32) of patients demonstrated radiographic evidence of healing of the discontinuity at the time of final follow-up, which could be attributed to the effect of central compression across the discontinuity achieved by acetabular distraction technique. However, in our series, there is not enough to make connections between bony callus formation and healing of the discontinuity in the X-ray.

Reconstruction of chronic pelvic discontinuity with extensive bone loss is fraught with difficulty and complications. Recently, Malahias et al. conducted a systematic review with regard to outcomes of acetabular reconstructions for the management of chronic pelvic discontinuity. Data showed that both CTAC and acetabular distraction techniques had a less than 5% all-cause revision rate at mean mid-term follow-up. Being the most commonly used method, cup-cage construct had an 8.1% revision rate for the acetabular component [18]. Thus, as can be seen, our results of the distraction technique are encouraging. This may also partially benefit from the additional use of augments and overlying cage. However, due to the short follow-up, the long-term survivalship of our technique remains to be observed.

The most common reason for reoperation after cup-cage or CTAC reconstruction was dislocation [18]. Especially in the use of CTAC, the relatively bigger construct provided more access to prosthetic impingement, injury of the superior gluteal nerve, and soft tissue dissection. Compared with cup-cage construct and CTAC, jumbo cup combined with acetabular distraction technique is associated with decreased surgical time and minimization of soft tissue stripping. We did not encounter any complications. It may have something to do with the small size of our series. The distraction technique did have some complications, such as femoral artery injury, bowel injury, sciatic nerve palsy, superficial infection, dislocation, and hematoma, as described by Sheth et al. [10]. However, the frequency of each complication was relatively lower compared with other methods [6, 8], especially the rate of dislocation and infection. It is worth noting that all neurovascular injuries occurred in their original cohort of patients undergoing acetabular distraction. At that time, implanted acetabular cup was selected to be 6 to 8 mm larger than the last reamer, and the acetabulum may have been overdistracted, resulting in stress transfer to the adjacent neurovascular structures [9].

Our method of distraction is more similar to their refined technique [10]. The appropriate-sized reamer was defined as allowing the surgeon to move the patient’s pelvis as a unit by moving the handle. And the acetabular component chosen for implantation was the same size as the last reamer. But we do not use a dedicated extra-acetabular distractor. We consider that osteoporosis is common among Asian patients with chronic pelvic discontinuity. A stiff device will probably cause further damage to the pelvis. To avoid inadequate distraction secondary to excessive discretion, we should ensure that pelvis can be moved as a whole, without micromotion.

In some cases of severe segmental bone defect, jumbo cup with acetabular distraction technique may be not enough to achieve stability. In our study, augment was used in two patients, and cup-cage construct was used in five patients. In fact, as a supplementary measure, the acetabular distraction technique was described by some authors [6, 19]. Namely, acetabular distraction technique itself can further secure the component by the fibrous recoil. However, perhaps under some circumstances, the acetabular reconstruction cannot do without the support of augments, and even an overlying titanium cage would be added to enhance the primary stability.

There are five limitations in our study. First, our series is relatively small due to the low incidence of these difficult cases. Second, with the same reason, there is no control group for comparison with other techniques. Third, the follow-up is not long enough, which makes the long-term durability of this technique pending. Fourth, the retrospective design of this study is a discernable limitation. Fifth, we did not assess intraobserver variability for assessing radiographs.

## Conclusions

Reverse reaming distraction is a feasible technique in treatment of chronic pelvic discontinuity. We observed only a single cup of aseptic loosening. Although the early results are encouraging, ongoing follow-up is required to determine the long-term prognosis in patients receiving this technique.

## Data Availability

All data generated or analyzed during this study are included in this published article.

## Abbreviations

THA: Total hip arthroplasty
HHS: Harris hip score
CTAC: Custom triflange acetabular component
3D: Three-dimensional
BMI: Body mass index
